# Evidence of Construct Validity of Computer-Based Tests for Clinical Reasoning: Instrument Validation Study

**DOI:** 10.2196/17670

**Published:** 2021-11-09

**Authors:** Tianming Zuo, Baozhi Sun, Xu Guan, Bin Zheng, Bo Qu

**Affiliations:** 1 Institute for International Health Professions Education and Research China Medical University Shenyang China; 2 Education Center for Clinical Skills Practice China Medical University Shenyang China; 3 Surgical Simulation Research Lab Department of Surgery University of Alberta Edmonton, AB Canada

**Keywords:** medical education, assessment, computer-based test, clinical reasoning, validity

## Abstract

**Background:**

Clinical reasoning (CR) is a fundamental skill for all medical students. In our medical education system, however, there are shortcomings in the conventional methods of teaching CR. New technology is needed to enhance our CR teaching, especially as we are facing an influx of new health trainees. China Medical University (CMU), in response to this need, has developed a computer-based CR training system (CMU-CBCRT).

**Objective:**

We aimed to find evidence of construct validity of the CMU-CBCRT.

**Methods:**

We recruited 385 students from fifth year undergraduates to postgraduate year (PGY) 3 to complete the test on CMU-CBCRT. The known-groups technique was used to evaluate the construct validity of the CBCRT by comparing the test scores among 4 training levels (fifth year MD, PGY-1, PGY-2, and PGY-3).

**Results:**

We found that test scores increased with years of training. Significant differences were found in the test scores on information collection, diagnosis, and treatment and total scores among different training years of participants. However, significant results were not found for treatment errors.

**Conclusions:**

We provided evidence of construct validity of the CMU-CBCRT, which could determine the CR skills of medical students at varying early stage in their careers.

## Introduction

Each year, several hundred thousand students enter medical school, all of whom need to equip themselves with the necessary health care skills and knowledge [1]. Since 2014, the vast majority of Chinese medical students attend a 5-year program after high school to earn a bachelor’s degree. Then, they work in a 1-year clinical internship before taking the nation’s standardized medical licensure exams. If successful, they may register as medical practitioners. Postgraduate training in medical specialties is standardized to 3-year programs with the final credential called Master of Medicine; this is now required of all clinical practitioners. In addition to learning a broad range of medical knowledge and practicing dexterity in hands, practitioners need to learn how to collect information from patients, process this information, and make accurate diagnostic decisions, similar to the expectations from a senior physician [2,3]. Clinical reasoning (CR) is a fundamental skill that separates medical personnel from other professionals. William Osler, a legendary pioneer medical educator, emphasized proper physical examination and diagnostic reasoning while maintaining the intimate physician-patient relationship. His teachings have resonated with generations of physicians [4]. Strictly speaking, CR refers to the procedure of collecting and integrating patient information from various sources to arrive at a diagnosis and management plan; it is usually case specific [5]. Every medical teaching institute makes a great effort to understand the nature of CR and improve strategies for teaching CR skills to health trainees [6]. However, the conventional methods that are used in our education system today are not optimal [7-11].

Traditionally, CR is taught in the classroom (didactic lecture) and by the patient’s side (clinical clerkship) [12-15]. A recent focus of integrating problem-based learning (PBL) has significantly improved the quality of CR education [6,12,16-18]. However, PBL relies heavily on the involvement and commitment of faculty instructors, which may not always be feasible [16,19]. Fidelity of case is also a problem compared to patient-side education [12]. Acquiring patient information by reading PBL cases from charts is quite a different experience than taking information directly from patients. Although instructors are making PBL cases in collaboration with clinicians, students still report a lack of case variety [17,20]. Creating sufficient clinical cases with clinical fidelity for CR training is a difficult task. Due to the above reasons, new technology is needed to improve our CR teaching.

In contrast to a paper- or lecture-based curriculum, computer-based CR training allows trainees to interactively take information from patients in a step-by-step process. There is also the possibility of accumulating a large volume of cases through international collaboration.

Currently, computer-based CR training can have different interfaces such as text, graphics, and animation [21]. The text-based CR training system is most widely used [22]. It is easy to create from clinical cases and deliver in the format of multiple choice questions or direct interface [23]. While medical images (including x-ray films, electrocardiograms, photos of lesions, etc) are required to give students more clinical information, graphic interface is also necessary. In several graphical models, illustrations of patients (in drawing or 2D pictures) can be used to create interactive experience for students when they collect information from patients [24]. Some computer-based CR training includes 3D animation or virtual reality technology to simulate the clinical scenario with high fidelity. However, the cost of creating 3D animation and virtual reality scenarios is much higher than the other computer-based CR models. It is difficult to create virtual patients without a team of technicians and instructional designers (Table 1).

**Table 1:** Types of computer-based clinical reasoning simulations and comparison.

Media	Advantage	Disadvantage
Text based	Relatively easy and rapid to develop; less expensive	Low level of fidelity
Graphic and animation based	Presents rich clinical evidence; moderate cost with enhanced fidelity	Replicates only part of clinical settings; low level of interactivity
Virtual reality	Combines highly sophisticated, life-like models with computer animations; can provide interactivity and feedback	Challenge to developers; often expensive

Sponsored by the National Medical Examination Center of China, China Medical University (CMU) started to developed computer-based CR training system in 2001. Educators and researchers at the Institute for International Health Professions Education and Research of CMU began to work with clinicians to develop cases for training CR skills and established the computer-based CR testing (CBCRT) system. Since 2002, CBCRT has been used as one part in the final comprehensive examinations of CMU to test the clinical skills of undergraduate students.

**Figure 1:**
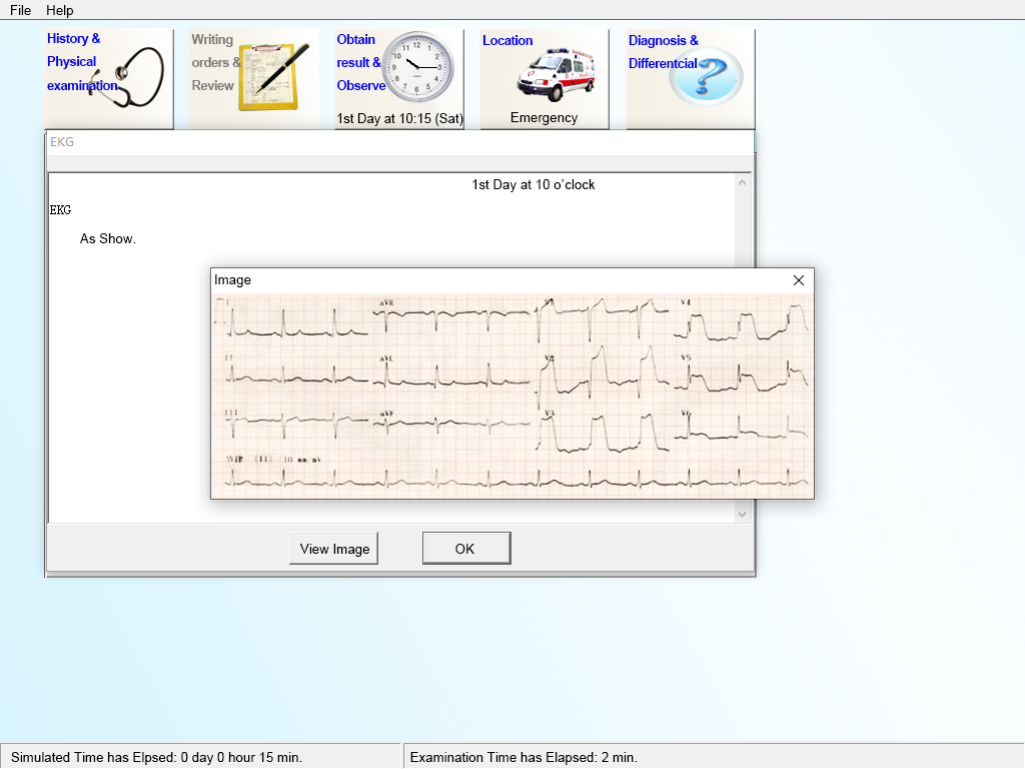
Screenshot displaying the main features of the China Medical University–computer-based clinical reasoning testing.

The CBCRT is composed of 5 interactive modules that allow students to interact with simulations to complete tasks: (1) history taking and physical examination, (2) writing orders and obtaining lab and medical imaging results, (3) reviewing obtained results, (4) working out diagnosis and differential diagnosis, and (5) observing the patient’s condition change at different phases and changing locations for managing different therapies. The main features of the CMU-CBCRT virtual patient are displayed in Figure 1.

To test the face validity of the CMU-CBCRT, we called a series of meetings with physicians and surgeons at which we screened and selected key information on each clinical topic for CR training. When a CR case was developed, our clinical team was surveyed to verify their clinical relevance. They then evaluated the interactive interface and rated their level of satisfaction.

To briefly summarize, the CBCRT provided clinical features of patients including history and physical and laboratory findings and then requires students to make a diagnosis as well as a treatment plan for the simulated patients. The CBCRT has also been welcomed by the examinees based on their positive feedback toward the system. Of 300 students surveyed using the questionnaire, 99.4% enjoyed participating in the CBCRT examination; 95.9% believed that the system accurately represents the real clinical environment; 72.5% agreed that the CBCRT is a better tool for teaching their clinical abilities. We can thus believe that the face validity of the CBCRT is satisfactory.

However, face validity is the weakest form of validity evidence. It can be only used at the primary stage of designing an assessment method [25]. We need to look into the structure of the CBCRT in detail to find more evidence of its validity, especially since there is a paucity of validity evidence for computer-based CR training [22]. In China, this study is the first of its type.

This study investigates construct validity of the CMU-CBCRT in medical trainees over 5 medical school. We hypothesize that the CMU-CBCRT will be able to determine CR level among different years of health trainees; specifically, senior trainees will achieve higher CBCRT scores than juniors.

## Methods

### Ethical Statement

Methods used for the project were reviewed and approved by the ethical review boards of the CMU (ERB 2016-027) and the 5 medical schools. Informed consent was obtained from each participant before they started the test with the CMU-CBCRT.

### Testing Sites

From November 24 to December 8, 2016, we implemented the CMU-CBCRT system in 5 collaborative medical schools: China Medical University, Fudan University School of Medicine, Sun Yat-sen University School of Medicine, Xuzhou Medical University, and Binzhou Medical College.

### Students

In China, medical students start their clerkships on the fifth year of medical training. The clinical training will continue for their 3 postgraduate years (PGYs). PGY 1 to PGY 3 is similar to the residency in North American medical school. We recruited students from fifth year undergraduates to PGY 3. The actual number of participants from each of 5 medical schools and their training years are shown as Table 2.

**Table 2:** Students from the 5 medical schools and their training years.

Schools	Fifth year medical student	PGY-1^a^	PGY-2	PGY-3	Subtotal
China Medical University	40	18	7	2	67
Fudan University School of Medicine	17	41	16	18	92
Sun Yat-sen University School of Medicine	12	28	20	12	72
Xuzhou Medical University	20	19	20	21	80
Binzhou Medical College	20	19	19	16	74
Total	109	125	82	69	385

^a^PGY: postgraduate year.

### Measures

**Figure 2:**
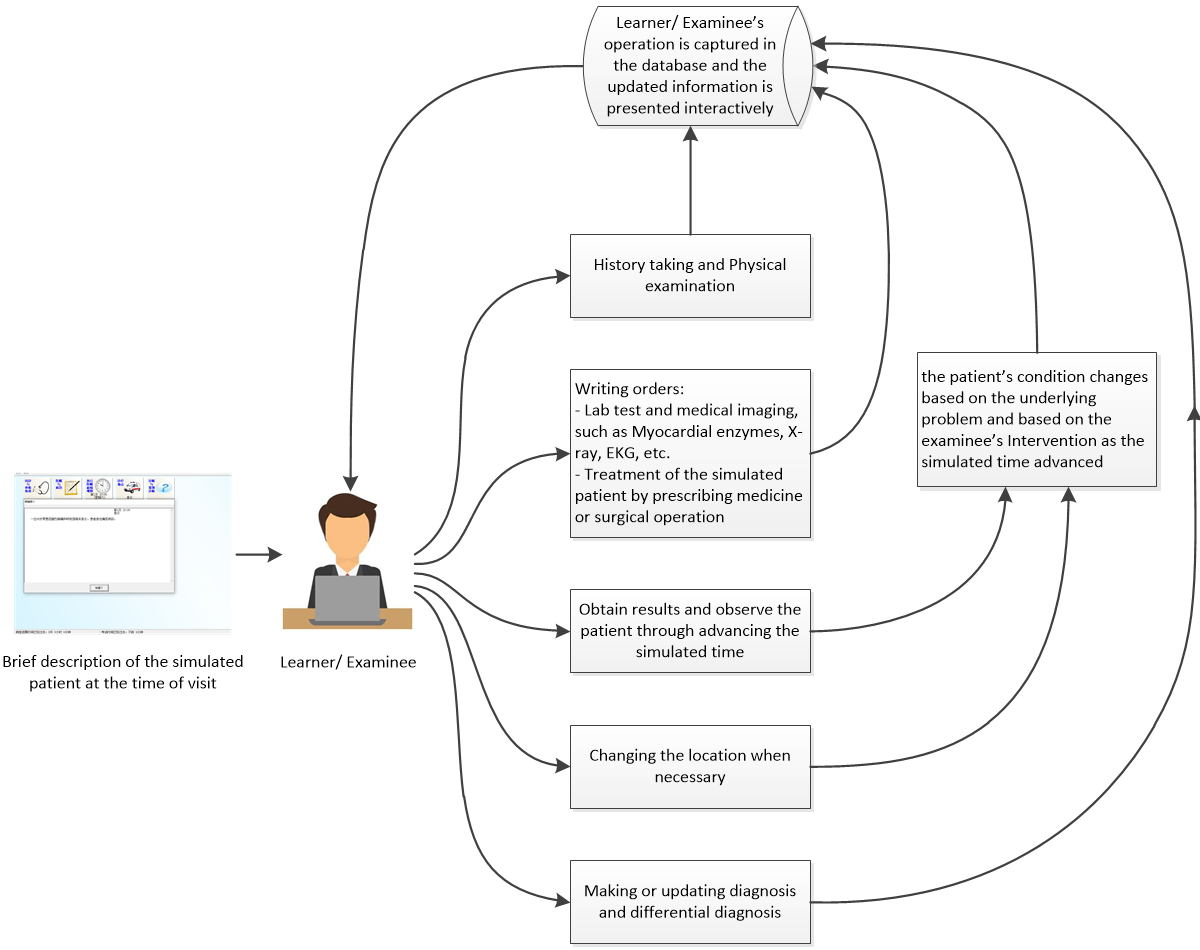
Outline of interaction flow through China Medical University–computer-based clinical reasoning testing.

Before testing, each student was asked to watch a 5-minute presentation and get familiar with the testing interface. Demographics and level of medical training were surveyed and recorded. The computer recorded participants’ typing and computer activity, including the typing and performance times. The interaction between a learner and how data are captured is displayed in Figure 2. Once completing the testing on CMU-CBCRT, the system calculated and recorded their total score by comparing the participants’ transaction list with the scoring scheme defined by the case developers committee (Multimedia Appendix 1). Subscores on these 4 different areas: information collection, diagnosis, treatment, and treatment error are computed and recorded as well (Multimedia Appendix 2).

### Statistical Model

The known-groups technique was used to evaluate the construct validity of the CBCRT by comparing the scores among the fifth year MD, PGY-1, PGY-2, and PGY-3 participants. Testing scores, including total and subtotal, were compared over the 4 training groups using a 1-way analysis of variance (ANOVA). Results were reported as mean and standard deviation. *P*≤.50 was considered a significant difference among testing groups.

## Results

### Total Score

**Figure 3:**
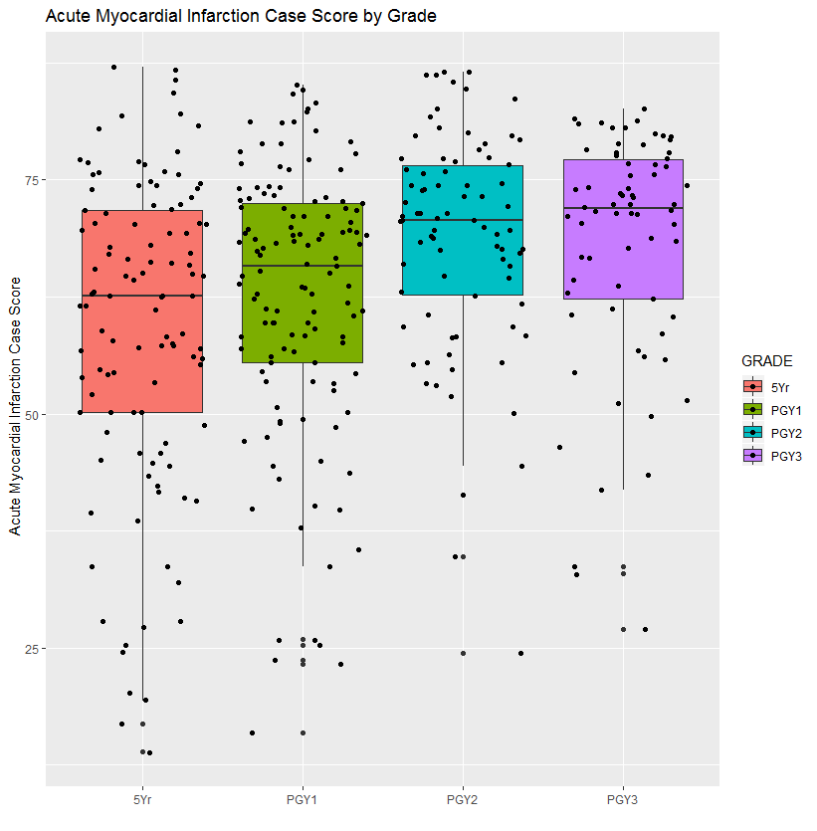
Total score of students over training years.

ANOVA revealed a group difference in total score among training levels (*P*<.001). As shown in Table 3 and Figure 3, the score of the fifth year MD students (59.01 [SD 16.68]) was significantly lower than the PGY-2 (68.68 [SD 11.76]) and PGY-3 (68.06 [SD 12.67]) students; the total score of PGY-1 students was also significantly lower than the PGY-2 and PGY-3 students.

**Table 3:** Students from the 5 medical schools and their training years.

Scores	Fifth year medical student, mean (SD)	PGY-1^a^, mean (SD)	PGY-2, mean (SD)	PGY-3, mean (SD)	*P* value
Information collection	43.42 (12.63)	46.70 (11.48)	49.73 (9.12)	51.38 (9.08)	<.001
Diagnosis	10.90 (4.74)	11.24 (4.97)	12.76 (3.90)	11.25 (4.22)	.034
Treatment	4.79 (3.81)	4.61 (3.36)	6.19 (3.73)	5.45 (3.72)	.013
Treatment error	–0.06 (023)	–0.04 (0.20)	0.00 (0.00)	–0.01 (0.12)	.13
Total	59.01 (16.68)	62.50 (14.45)	68.68 (11.76)	68.06 (12.67)	.001

^a^PGY: postgraduate year.

### Subscore

**Figure 4:**
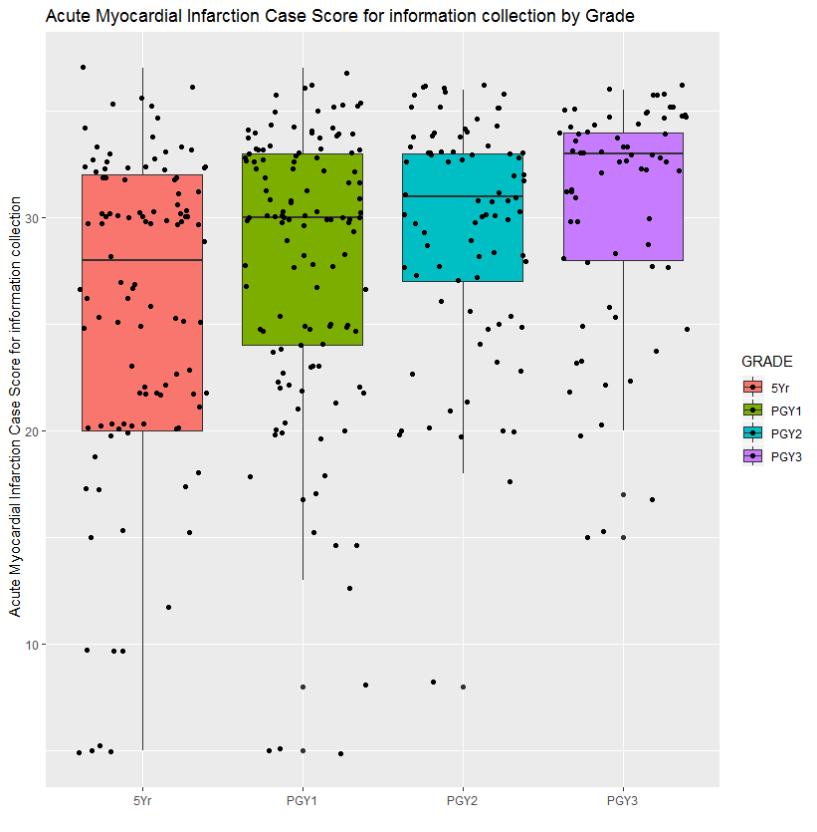
Subscore for information collection of students over training years.

**Figure 5:**
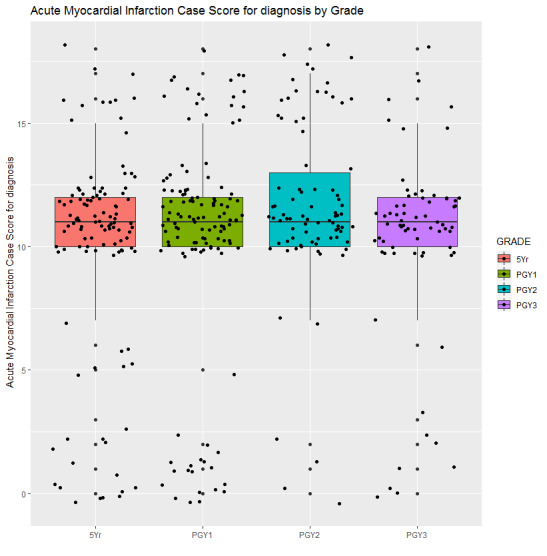
Subscore for diagnosis of students over training years.

**Figure 6:**
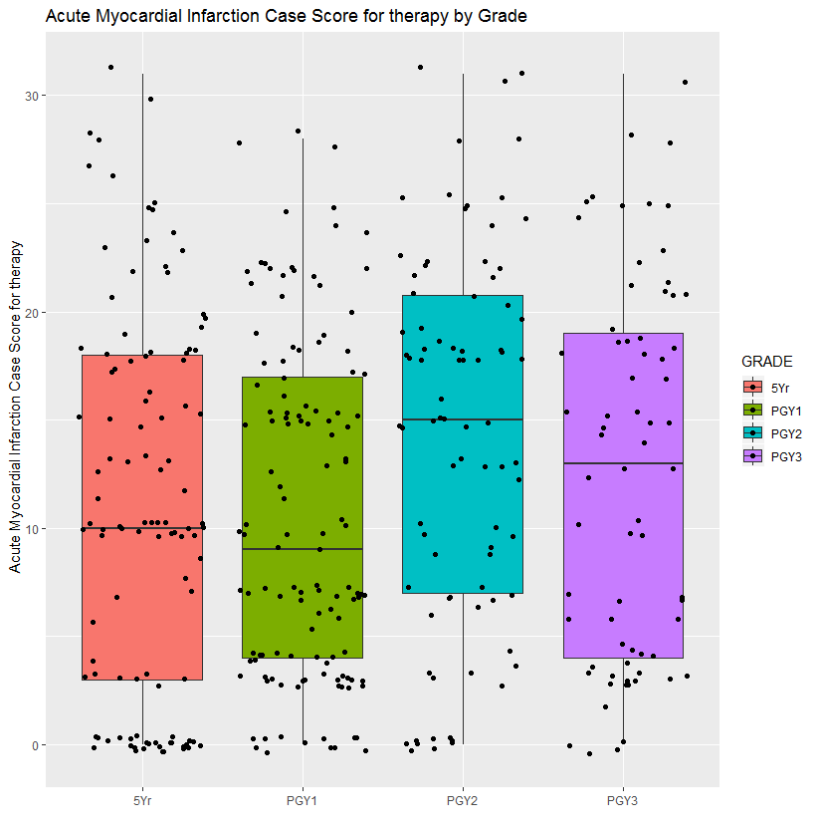
Subscore for treatment of students over training years.

ANOVA revealed group differences by training level between information collection (*P*<.001), diagnosis (*P*=.03), and treatment (*P*=.01) scores, but not on treatment error (*P*=.13) score. As shown in Figure 4, the information collection scores of the fifth year MD students (43.42 [SD 12.63]) were significantly lower than the PGY-1 (46.70 [SD 11.48]), PGY-2 (49.73 [SD 9.12]), and PGY-3 (51.38 [SD 9.08]) students; information collection scores of PGY-1 students were also significantly lower than the PGY-3 students. As shown in Figure 5, the diagnosis scores of the fifth year MD (10.90 [SD 4.74]), PGY-1 (11.24 [SD 4.97]), and PGY-3 (11.25 [SD 4.22]) students were significantly lower than the PGY-2 (12.76 [SD 3.90]) students. As shown in Figure 6, treatment scores of the fifth year MD (4.79 [SD 3.81]) and PGY-1 (4.61 [SD 3.36]) students were significantly lower than the PGY-2 (6.19 [SD 3.73]) students.

## Discussion

### Principal Findings

Before applying an assessment tool for use with medical students, we must obtain evidence for the instrument’s reliability and validity [26-28]. Providing evidence of the validity of CBCRT will help the test management organization understand the effectiveness of the test from a broad and comprehensive perspective, clarify the aspects that the CBCRT can and cannot measure, and hence, allow for its continuous improvement. This is the goal of our current study. Our hypothesis was supported by the results obtained; specifically, senior students displayed higher testing scores than junior students (Table 3, Figure 1). In other words, the CMU-CBCRT is able to determine CR skills over different levels of medical education, especially in the early stage of the students’ medical careers.

Looking specifically into the 4 categories of skills that we tested, we found that the most significant differences were revealed in the information collection, diagnosis, and treatment scores among junior and senior medical students. This was as predicted. With years of training, their experience and ability to clinically reason are improving, and as a result, they performed better on the information collection, diagnosis, and treatment, as well as the total CBCRT score. This further suggests that the CMU-CBCRT can determine the CR skills of students at varying levels.

We also carefully studied and analyzed why there were no significant differences in treatment error scores among the 4 training groups. For a simulated case of myocardial infarction, we can observe from the test result the challenge faced by participants who have never experienced this form of examination before. When the passing score was set at 60%, the average score in this case (59.01 [SD 16.68]) did not pass. The choice of wrong treatment is a negative item in the scoring system, so the item writing expert is very cautious in formulating the scoring standard. Only behavior that caused extreme consequences resulted in points being deducted, and the weight was set at a very low level (ie, –1%). In this test, we observed that treatment error behavior happened more with junior students than senior students, although without statistical significance.

In the absence of an available gold standard for measuring CR, evidence for construct validity is sought after in this area of research. This is an ongoing process, in which the skill measured by the assessment tool is linked to some other attribute by a hypothesis or construct. With the development of validity theory, the validity concept has a new connotation and forms a method based on multilevel evidence [22]. Validity is no longer an attribute of the measurement tool itself but rather the extent to which the evidence collected supports the interpretation, inference, and decision making of the test score [27,29].

With the positive evidence presented, we should still be aware that validity verification is a dynamic process [27] and no education instrument is 100% effective [27]. Even if the evidence indicates that the validity of a course test is significant, the validity study must continue along with the development of the CBCRT system. There are still many problems to be solved, such as the setting of the evidence framework for the specific test validity, determination of the validity criteria, feasibility of the evidence collection method, and quantification of evidence data. This will require in-depth discussion by future researchers. We aim for constant examination of these issues in the process of developing a reliable and valid CR training model. In the future, we would include more simulated cases with a wide range of case difficulties and distribute CMU-CBCRT to more students to increase sample size. We would then carefully collect data on student performance and feedback. We also plan to add graphics and animation to enhance the interface design.

### Limitations

However, there were some limitations in our study to its generalizability. First, the respondents of the research were from only 5 medical institutions in China. Second, the findings of our study were limited by the representativeness and scale of the study population.

### Conclusions

We provided evidence of construct validity of the CMU-CBCRT. It is able to determine CR skills over different levels of medical education, especially in the early stage of the students’ medical careers.

## Appendix

Multimedia Appendix 1 Scoring sheet and scoring rubrics.

Multimedia Appendix 2 China Medical University–computer-based clinical reasoning testing data.

## Abbreviations

ANOVA: analysis of variance
CBCRT: computer-based clinical reasoning testing
CMU: China Medical University
CR: clinical reasoning
PBL: problem-based learning
PGY: postgraduate year
